# The Impact of Heart Failure Chronic Treatment Prior to Cardiac Transplantation on Early Outcomes

**DOI:** 10.3390/medicina60111801

**Published:** 2024-11-03

**Authors:** Dragos-Florin Baba, Horatiu Suciu, Calin Avram, Marius Mihai Harpa, Mircea Stoian, Diana-Andreea Moldovan, Laurentiu Huma, Gabriel Rusu, Tunde Pal, Alina Danilesco, Adina Stoian, Anca-Ileana Sin

**Affiliations:** 1 Department of Cell and Molecular Biology, George Emil Palade University of Medicine Pharmacy, Science, and Technology of Targu Mures, 540142 Targu Mures, Romania; dragos-florin.baba@umfst.ro (D.-F.B.); laurentiu.huma@umfst.ro (L.H.); ileana.sin@umfst.ro (A.-I.S.); 2 Emergency Institute for Cardiovascular Diseases and Transplant, 540136 Targu Mures, Romania; horatiu.suciu@umfst.ro (H.S.); mariusharpa@gmail.com (M.M.H.); diana.moldovan@umfst.ro (D.-A.M.); gabriel.rusu@ibcvt.ro (G.R.); 3 Department of Surgery, George Emil Palade University of Medicine, Pharmacy, Science and Technology of Targu Mures, 540142 Targu Mures, Romania; 4 Department of Medical Informatics and Biostatistics, George Emil Palade University of Medicine, Pharmacy, Science and Technology of Targu Mures, 540142 Targu Mures, Romania; 5 Department of Anesthesiology and Intensive Care, George Emil Palade University of Medicine, Pharmacy, Sciences and Technology of Targu Mures, 540139 Targu Mures, Romania; mircea.stoian@umfst.ro; 6 Targu-Mures County Hospital, 540072 Targu Mures, Romania; alinka942@yahoo.com; 7 Department of Family Medicine, George Emil Palade University of Medicine, Pharmacy, Science and Technology of Targu Mures, 540142 Targu Mures, Romania; 8 Department of Internal Medicine V., George Emil Palade University of Medicine, Pharmacy, Sciences and Technology of Targu Mures, 540136 Targu Mures, Romania; 9 Department of Pathophysiology, George Emil Palade University of Medicine, Pharmacy, Sciences and Technology of Targu Mures, 540136 Targu Mures, Romania; adina.stoian@umfst.ro

**Keywords:** cardiac transplantation, complications, Carvedilol, Ramipril, Spironolactone, mortality

## Abstract

*Background and Objectives*: Cardiac transplantation represents the option for patients with end-stage heart failure (HF), providing the best survival rate. However, the postoperative complications of transplant patients remain a challenge for clinicians. The objective of our study was to evaluate the effect of preoperative chronic HF treatment on the occurrence of in-hospital complications. *Materials and Methods*: We retrospectively included a total of 50 patients who underwent cardiac transplantation between January 2011 and December 2023 from the Emergency Institute for Cardiovascular Diseases and Transplantation of Targu Mures. We correlated the preoperative chronic HF treatment with the postoperative complications by Spearmen’s correlation coefficient, respectively. With logistic regression, the associations between the treatment and specific complications were determined. *Results*: Significant negative correlations were found between Carvedilol treatment with 2-month mortality (r = −0.30; 95% CI: −0.53–−0.02; *p* = 0.03), Ramipril with hospital stay (r = −0.38; 95% CI: −0.60–-0.12; *p* < 0.01) and intensive care unit (ICU) stay (r = −0.37; 95% CI: −0.59–−0.11; *p* = 0.01), and Spironolactone usage with hospitalization duration (r = −0.28; 95% CI: −0.52–−0.01; *p* = 0.04). Furthermore, Carvedilol treatment represented a protective factor against early acute kidney injury (AKI) (OR: 0.22; 95% CI: 0.05–0.91; *p* = 0.03). Spironolactone treatment was a protective factor against AGR (OR: 0.12; 95% CI: 0.02–0.66; *p* = 0.01) treatment, in contrast to angiotensin-converting enzyme inhibitor (ACEI) therapy (OR: 5.30; 95% CI: 1.03–27.17; *p* = 0.04). *Conclusions*: Pre-transplant Carvedilol treatment was negatively correlated with the 2-month mortality rate. Ramipril and Spironolactone therapy were negatively correlated with hospitalization duration, and Ramipril was additionally correlated with ICU stay. Moreover, Carvedilol therapy represented a protective factor against early AKI. Pre-transplant Spironolactone was associated with lower event rates of AGR, in contrast to ACEI treatment. Prospective studies with larger cohorts are needed in order to draw drastic conclusions.

## 1. Introduction

Cardiac transplantation provides the best survival benefit for patients diagnosed with end-stage heart failure (HF). Now, the median survival of heart-transplanted patients has reached more than 12 years. Though, several limitations, such as the narrowed availability of graft and postoperative graft dysfunction, together with acute/chronic graft rejection and non-cardiac complications, represent major challenges of heart transplantation [1].

In patients undergoing surgery, the perioperative inflammatory response is associated with postoperative complications, independently from the surgical factors [2]. There have been significant associations between the preoperative inflammatory status and the postoperative infections reported [3]. In cardiac surgery, different factors create an inflammatory reaction, leading to ischemia-reperfusion injury and systemic inflammatory response [4]. We have previously shown that the cardiac patients of our institute have associations between preoperative inflammatory markers and the apparition of postoperative complications [5,6].

In heart transplantation, there has been reported multiple risk factors associated with postoperative mortality, such as very young/advanced recipient age, ventilator support at the time of transplantation, kidney dysfunction, decreased cardiac output, elevated pulmonary vascular resistance, longer donor ischemic time, advanced age of the donor and, donor and recipient without blood type O [7]. The donor’s heart preservation time of more than 5 h poses a risk in receipt survival, which is amplified by the need for preoperative dialysis and extracorporeal membrane oxygenation (ECMO), diagnosis of ischemic cardiomyopathy (CM) causing end-stage HF, and the use of the O blood type [8]. Moreover, the need for mechanical circulatory support before transplant and Amiodarone exposure were associated with high-inotrope administration, and a prolonged ischemic time with several perioperative transfusions was associated with primary graft dysfunction [9].

Early postoperative echocardiographic markers, such as the tricuspid regurgitation, tricuspid annular plane systolic excursion-to-systolic pulmonary artery pressure ratio (TAPSE/sPAP), and left ventricular relaxation index, represent factors involved in the occurrence of post-transplant complications and patient survival [10,11,12]. Myocardial velocity and deformation imaging showed promising results for the early detection of post-transplant cardiac allograft vasculopathy and timed coronary angiographies in order to reduce the number of routine endomyocardial biopsies [13].

We have previously observed the fact that preoperative chronic Statin treatment before cardiac transplantation, more precisely Atorvastatin, reduced the overall rate of postoperative complications but at the same time contributing to an elevated risk of early-postoperative acute kidney injury (AKI) and newly developed diabetes mellitus (DM). Furthermore, high C-reactive protein (CRP) levels represented risk factors, with Atorvastatin administration being independently correlated with lower levels of this inflammatory marker [14]. A meta-analysis showed that the use of Statins after a heart transplant improves the survival of recipients, preventing fatal rejection episodes and reducing the terminal cancer risk and coronary vasculopathy incidence [15]. 

The impact of these HF medications administered before heart transplant on immunosuppressive treatment has not been investigated before. The beneficial/detrimental effects of these drugs might have an impact on immunosuppressive therapy management, interacting with the incidence of early acute graft rejection (AGR) and secondary dangerous effects, such as postoperative infections, AKI, and apparition of DM. The purpose of our study was to evaluate the effect of preoperative chronic HF treatment in the occurrence of 2-month complications regardless of cause, hospital stay, duration of inotrope/vasopressor therapy, intensive care unit (ICU) stay, and 2-month mortality rate. Secondly, we assessed the impact of the pre-transplant HF treatment on specific complications, such as the apparition of in-hospital AGR, early postoperative AKI, newly diagnosed DM, or atrial fibrillation (AFib). Patient management after the transplant follows guidelines that are uniform in the center where the surgery took place, making the patients’ postoperative evolution predictable at a certain level. The influence that preoperative management on postoperative complications and mortality, considering the inhomogeneous therapies that patients receive, possesses a potential optimal algorithm for patients waiting for heart transplants.

## 2. Materials and Methods

This study was performed retrospectively, including patients from the Emergency Institute for Cardiovascular Diseases and Transplantation, Targu Mures, Romania. A total of 51 patients underwent cardiac transplantation between January 2011 and December 2023. After applying the inclusion criteria, one patient was excluded because of the lack of data. In accordance with the Declaration of Helsinki, this study’s protocol was validated by the ethics committee of the Cardiovascular and Transplantation Emergency Institute of Targu Mures. All patients included in this research had their informed consent signed at admission.

**Figure 1:**
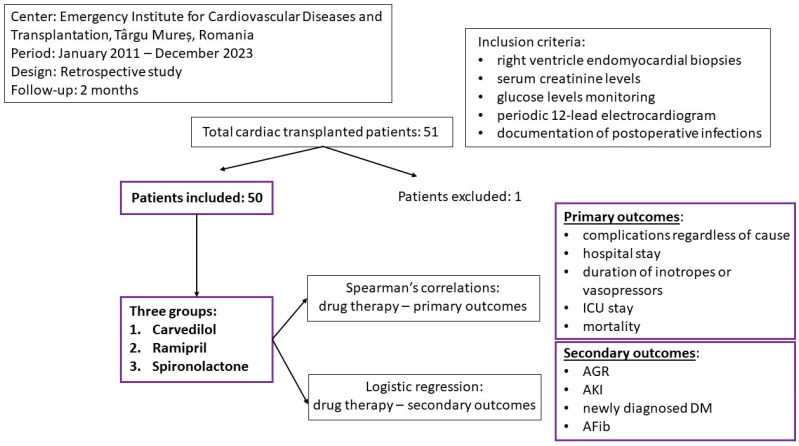
Study design.

The inclusion criteria were represented by the postoperative right ventricle endomyocardial biopsies, consecutive measurements of serum creatinine levels in the first 72 h after transplant, glucose level monitoring, periodic 12-lead electrocardiogram, and documentation of postoperative infections during the hospital stay. We assessed the hospital duration, duration of inotrope/vasopressor administration, and ICU stay, and using the internet system of the Emergency Institute for Cardiovascular Diseases and Transplantation of Targu Mures, we determined the 2-month survival rate of the cohort. Previous chronic drug treatment was documented in the patient’s files (Figure 1).

Our cohort was divided into 3 different groups based on the specific treatment of the HF (beta blockers (BBs)—Carvedilol, angiotensin-converting enzyme inhibitors (ACEIs)—Ramipril, mineralocorticoid receptor antagonists (MRAs)—Spironolactone) and was investigated in terms of the drug daily dosage, age, gender, weight, body surface area (BSA), body mass index (BMI), blood type, ischemic etiology of the CM, and previous diagnosis of DM. Secondly, we divided the cohort into two groups based on the apparition of postoperative complications regardless of cause. Taking into consideration the reduced number of patients in our cohort with chronic treatment with sodium-glucose transport protein 2 inhibitor (SGLT2i) (5 patients out of 50), we have decided not to include this therapy in further analysis.

Primary endpoints were represented by the 2-month complications regardless of cause, hospital stay, duration of inotrope/vasopressor therapy, ICU stay, and 2-month mortality rate. For the complications regardless of cause, we counted the presence of postoperative AGR, early AKI, newly diagnosed DM, or AFib. The secondary endpoints were the specific in-hospital post-transplant complications, such as AGR, early AKI, newly diagnosed DM, or AFib (Figure 1).

Data processing was performed using MedCalc version 19 (MedCalc Software Ltd., Ostend, Belgium) for the quantitative data, determining the mean values, standard deviation (SD), and percentages of each group. Firstly, we compared the cohort by the presence/absence of complications regardless of cause, and the test chosen for evaluating the normal level distribution was the Shapiro–Wilk test. The comparison was made using Student’s *t*-test for parametric data and the Mann–Whitney U test for the non-parametric data. In the case of categorical values, the chi-square test was applied. We correlated the preoperative chronic HF treatment with the primary endpoints by Spearmen’s correlation coefficient. Lastly, using logistic regression, we evaluated the associations between chronic therapy before the transplant and secondary endpoints with a 0.05 significance threshold.

## 3. Results

The cohort’s mean age was 41.5 years (SD = 14.1 years), with a mean BMI of 23.6 kg/m^2^ (SD = 4.9 kg/m^2^), and eight (16%) of them were females. The majority of patients were Rh positive (90.0%; 45 out of 50 individuals), and the most frequent blood group was A type (50.0%). The diagnostic of ischemic CM was seen in 11 individuals (22.0%) prior to cardiac transplantation. A total of 38 patients (76.0%) had a previous treatment with BBs, 19 patients (38.0%) had ACEIs, and 46 patients (92.0%) had MRAs. Seven patients (14.0%) were treated with Sacubitril/Valsartan, and five were treated with SGLT2i (10.0%) (Table 1).

A total of 26 patients (52.0%) had chronic treatment with Amiodarone, 19 (38.0%) had Ivabradine, and 10 (20.0%) had Digoxin. In terms of the pre-transplant-specific HF drug therapy, 32 patients (64.0%) were treated with Carvedilol, 13 patients (26.0%) with Ramipril, and 41 patients (82.0%) with Spironolactone. The mean hospital stay was 57.5 days (SD = 62.7 days), being on inotrope/vasopressor administration for 8.4 days (SD = 12.4 days), with a mean ICU stay of 49.9 days (SD = 59.2 days) (Table 1).

There were significant differences in terms of positive Rh (89.5% versus 91.7%; *p* < 0.01), mean hospitalization duration (64.0 days vs. 36.8 days; *p* = 0.03), and mean ICU stay (56.4 days vs. 29.5 days; *p* = 0.01) in the favor of the patients who did not present any complications counted in the primary outcome (Table 1).

Complications of any cause were experienced by a total number of thirty-eight patients (76.0%), from which eleven (22.0%) had AGR, twenty-eight (56.0%) developed early postoperative AKI, thirty (60.0%) had newly diagnosed DM, ten (20.0%) had postoperative AFib, and nine individuals (18.0%) presented criteria of sepsis. The 2-month mortality of the cohort was 10% (five out of fifty patients).

Carvedilol’s mean daily dosage was 19.1 mg (SD = 12.3 mg), 5.2 mg (SD = 2.4 mg) for Ramipril, and 61.0 mg (SD = 29.1 mg) for Spironolactone. The rate of AGR of the Spironolactone group was 14.6% (6:41) compared with 55.6% of the non-Spironolactone group (5:9). A percentage of 43.8% from the group with Carvedilol (14:32) experienced AKI compared to 77.8% from the non-Carvedilol group (14:18). For hospitalization duration, the Spironolactone group had a mean stay of 53.9 days (SD = 64.8 days) compared with 73.6 days (SD = 51.8 days) in the non-Spironolactone group, and a mean duration for the Ramipril group was 40.6 days (SD = 40.6 days) compared with 63.4 days (SD = 68.3 days) in the non-Ramipril group. In terms of the ICU stay, the mean duration for the Ramipril group was 34.0 days (SD = 27.1 days) versus 55.5 days (SD = 66.4 days) in the non-Ramipril group. In the non-Carvedilol group, 2-month mortality was 22.2% (4:18) compared with 3.1% (1:32) in the Carvedilol group (Table 2).

Significant negative correlations were found between the preoperative chronic treatment with Carvedilol and the 2-month mortality (r = −0.30; 95% CI: −0.53–−0.02; *p* = 0.03), Ramipril with hospital stay (r = −0.38; 95% CI: −0.60–−0.12; *p* < 0.01), Ramipril and ICU stay (r = −0.37; 95% CI: −0.59–−0.11; *p* = 0.01), and Spironolactone usage and the hospitalization duration (r = −0.28; 95% CI: −0.52–−0.01; *p* = 0.04). No significant correlations were determined between the drug therapy and the composite of complications regardless of cause nor the duration of inotrope/vasopressor postoperative administration (Table 3).

In logistic regression, Carvedilol treatment represented a protective factor against early AKI (OR: 0.22; 95% CI:0.05–0.91; *p* = 0.03). Regarding AGR, MRAs (OR: 0.05; 95% CI: 0.01–0.67; *p* = 0.02) and Spironolactone (OR: 0.12; 95% CI: 0.02–0.66; *p* = 0.01) treatment were protective factors, in contrast to ACEI therapy, which has been associated with a higher risk of developing in-hospital AGR (OR: 5.30; 95% CI: 1.03–27.17; *p* = 0.04). No significant associations were observed in terms of newly developed DM and postoperative AFib (Table 4).

## 4. Discussion

The pleomorphic effects of the chronic HF treatment before cardiac transplantation might have a beneficial impact on postoperative evolution. The interactions of these HF drug treatments with immunosuppressive therapy in heart transplants is still unclear, as well as their effects on the beneficial/secondary adverse effects. We found significant negative correlations between the preoperative treatment with Carvedilol and the 2-month mortality rate. Preoperative Ramipril and Spironolactone treatment were weakly correlated with hospitalization duration, and Ramipril was correlated with ICU stay. No statistical correlations were found between the chronic treatment and the complications regardless of cause. Meanwhile, regarding specific postoperative complications, Carvedilol treatment was a protective factor against early AKI. MRA therapy, specifically Spironolactone, was associated with lower rates of in-hospital AGR, in contrast to ACEI treatment.

Carvedilol treatment might reduce inflammation, which was proven by the decreased level of high-sensitivity C-reactive protein (hsCRP) in patients with chronic HF. The BRIGHT-D trial showed anti-inflammatory effects in chronic HF of BBs, such as Bisoprolol and Carvedilol. Bisoprolol revealed better results than Carvedilol in reducing inflammation, but Carvedilol was better for reducing oxidative stress [16]. In mice with viral myocarditis, Carvedilol was shown to be more potent than metoprolol in ameliorating myocardial lesions based on the impact on the balance between pro- and anti-inflammatory cytokines [17]. Treatment with Carvedilol might also be beneficial in other inflammatory diseases, such as arthritis, rosacea (chronic skin disorder), and hepatic encephalopathy [18,19,20]. Moreover, Carvedilol could have an impact on modulating the granulocyte-macrophage colony-stimulating factor, lowering interleukin-10 (IL-10) production and representing a key factor in regulating inflammatory reactions [21].

Aldosterone, a mineralocorticoid that modulates the volumes and concentration of blood electrolytes, has been associated with arterial hypertension and major cardiovascular events, possibly through the pro-inflammatory effects, increasing the reactive oxygen species levels [22]. However, the inflammatory information about Spironolactone is controversial. In patients with diabetes, Spironolactone reduces blood pressure and urinary albumin, improving fibrosis and inflammation [23]. Apparently, Spironolactone did not have any impact on hsCRP levels at one year, with high values of this marker being a risk factor in HF individuals [24]. The TOPCAT trial evidenced potential impacts on markers of cardiac wall stress or filling pressures, such as B-type natriuretic peptide (BNP) and N-terminal-pro-B-type natriuretic peptide (NT-proBNP), in patients treated with spironolactone, with chronic HF with preserved ejection fraction [25]. Although, Spironolactone inhibits pro-inflammatory cytokines, such as the tumor necrosis factor-alpha (TNF-alpha), IL-6, IL-1, and interferon-gamma (IFN-gamma) production, inhibition produced at the transcriptional level, independently of the anti-mineralocorticoid and anti-androgen activities [26].

### 4.1. Graft Dysfunction

Although heart transplantation arose as a safeguard for end-stage HF patients back in the 1960′s, short- and long-term complications still represent a challenge for both physicians and patients [27,28]. However, thinking about the long-term results, due to technical and pharmaceutical developments and selection criteria, the survival rate reached 50% at 10 years, 30% at 15 years, and 17% at 20 years in the United States [29]. One of the causes of early mortality post-transplant is primary graft dysfunction characterized by left or right ventricular dysfunction, and even biventricular dysfunction can be possible. The incidence of primary graft dysfunction was reported in 7% and denotes a 30% mortality in the first month. Other common causes of death in the first month are multiorgan failure and sepsis. Several risk factors were suggested in previous studies, such as ischemia during organ recovery, injuries or unrecognized diseases of the donor’s heart, and reperfusion lesions [30]. Later, allograft vasculopathy is recognized as the most important cause of death [1]. A better survival rate at one year in patients who were transplanted for ischemic etiology compared to dilated CM was suggested [1]. In 50 patients, the 2-month mortality of our sample was 10%, with a lower rate in patients with Carvedilol medication prior to transplant. Post-transplant administration of BBs has contradictory results, as in a study, one-month survival was not improved by BB administration in 380 patients [31] compared to the study by Ciarka et al. [32], who reported a higher mortality rate in patients without BBs. Nevertheless, the use of BBs after heart transplantation has some rationale based on the fact that there is evidence that an increased heart rate may contribute to higher mortality and allograft vasculopathy [33,34]. However, although heart transplant improves survival rates in end-stage HF patients, pharmacological treatment still is essential before and after the intervention. Based on our results, HF guideline-recommended drugs [35] prior to heart transplantation improved post-transplant results.

### 4.2. Acute Graft Rejection

The success of cardiac transplantation has been obtained by the development of improved immunosuppressive therapies. The diagnosis of graft rejection results from the usage of endomyocardial biopsies [36]. The AGR threat is continuously present in the everyday lives of cardiac transplanted recipients, resulting in a decrease in overall psychological well-being [37]. There is evidence that repeated cellular graft rejections increase cardiac inflammation, resulting in an elevated risk of allograft vasculopathy [38]. The humoral AGR poses a higher incidence in women, and it is associated with hemodynamic compromise, accelerated allograft vasculopathy and death [39]. Moreover, late humoral rejection has a poor prognosis, despite aggressive immunosuppressive therapies, and it is conducive to allograft vasculopathy [40].

### 4.3. Acute Kidney Injury

AKI is one of the most frequent heart transplantation-related complications. In a meta-analysis, the presence of AKI in patients who benefited from cardiac surgery was 20% [41]. The rates of AKI after heart transplant have a wider range, with some studies reporting even a 78% percentage in heart transplant receipts [42]. Our results, with a rate of 56% of AKI, were in concordance with similar studies, in which among the risk factors identified were longer cardiopulmonary bypass time, transfusions, higher central venous pressure, longer duration of inotropic and vasopressor agents, right ventricle function, diabetes mellitus, and higher BMI [42,43,44]. On the other hand, AKI was associated with a higher mortality rate in patients’ primary graft dysfunction in the first year [44], and it is a risk factor for AGF too [45]. Early renal replacement therapy improves mortality and reduces ICU and hospital stay [46]. Ideally, the prevention of post-op AKI is warranted. From the investigated non-pharmacological prevention strategies, cardiopulmonary bypass with pulsatile flow resulted in a lower rate of AKI, and it was also associated with fewer days of hospitalization and ICU stay; however, it had a wide heterogeneity. In contrast, tight glycemic control did not affect the prevention of AKI [41]. However, AKI following a heart transplant has a more complex background; a higher proportion of patients suffer from AKI, which negatively influences further outcomes [44,47]. In AKI, the most studied drugs are those that block the renin–angiotensin–aldosterone system. However, in terms of cardiac surgery, further research is needed [48]. We found no significant association between AKI and the blockers of the renin–angiotensin–aldosterone system. The effect of BB therapy on kidney function in patients following heart transplantation is understudied. In our study, BB therapy with Carvedilol was associated with lower rates of AKI in patients after heart transplantation. In a meta-analysis, Carvedilol added to N-acetyl cysteine reduced AKI after cardiac surgery [48]. BB therapy is a guideline-recommended first-line drug in HF patients, which improves mortality and hospitalization [35]. Nevertheless, these beneficial effects of BBs after heart transplantation were not proven in prior studies [31]. Moreover, a transient increase in creatinine was observed in HF patients on Carvedilol, indeed without the risk of severe AKI [49]; thus, it can be used without further concerns [50].

### 4.4. Development of Diabetes Mellitus

Newly diagnosed DM is commonly seen after heart transplant, with an incidence of more than 20% of patients in the first year post-transplant. Glucagon-like peptide-1 receptor agonists and SGLT2i might address the secondary hyperglycemia mechanisms of immunosuppression therapy with cardiovascular and renal benefits [51,52]. Post-transplant DM is associated with poor postoperative outcomes, such as infection, renal injury, microvascular disease, re-transplantation, and mortality. Early post-transplant hyperglycemia risk factors include recipient and donor age, BMI, infections, and chronic inflammation, together with post-transplant immunosuppression therapy [53]. In post-transplant hyperglycemia, early insulin administration is recommended [53]. Hecking et al. [54] demonstrated renal transplantation and that insulin therapy in the early post-transplant period decreased the chances of immunosuppression treatment damage on beta-pancreatic cells.

### 4.5. Atrial Fibrillation

AFib has a higher prevalence after cardiac surgery, including heart transplantation, and increases the rates of complications and mortality [55]. Although pulmonary vein isolation and denervation of the transplanted heart might protect from AFib [56], it has numerous risk factors that are not necessarily limited to cardiovascular-related ones [57]. The estimated incidence of post-transplant AFib in a 5393 transplanted patient was 10% [58]. In our study, 20% of patients presented AFib after transplantation, similar to Ferretto et al. [56]. Even though BBs along with amiodarone reduce AFib after cardiac surgery [55], in line with Darche et al. [59], we found no significant association between the administered medication and the presence of this arrhythmia. Prior research has demonstrated that pre-transplant AFib increases the risk of post-transplant AFib [59], and also AGR has an impact on AFib incidence in the first two months [56], though AFib-related and antithrombotic therapy-related complications, like bleeding or stroke, are low, as well as hospitalization for AFib [60]. On the contrary, another study reported increased mortality in patients with AFib in the first month compared to patients without AFib in this period, but the same was present in pre-existing AFib prior to heart transplantation [59]. In terms of the AFib cardioversion approach, in patients with HF, catheter ablation seems to have benefits on survival, improving the left ventricle ejection fraction (LFEF) and their quality of life [61].

### 4.6. Limitations

The limitations of our study must be pointed out. First of all, the retrospective model of our research is conducive to a number of limitations, such as losses to follow up, information bias, and losses of important data [62]. Nevertheless, retrospective studies can have their strengths but often are considered to be inferior to prospective, randomized, and controlled clinical trials [63]. Secondly, the small number of patients in our cohort might cause the overestimation of the OR, with a large CI derived from the logistic regression [64]. Unfortunately, in terms of transplantation, Romania holds a low place in the ranking of countries in the European Union [65]. In our country, organ donation still displays ethical challenges at personal and community levels, which is also caused by Romania’s bureaucracy [66,67]. Regarding liver transplantation in Romania, the national program constantly evolved over time, resulting in lower mortality rates of the patients from the waiting list [68]. Lastly, the effects of SGLT2i were not investigated and were secondary to the low number of patients benefiting from this treatment. The fact that patients with heart transplants might experience signs and symptoms of HF with preserved ejection fraction (HFpEF) is known, with SGLT2i proving to improve the right ventricle performance [69] with theoretical potential benefits in heart transplanted patients. Future campaigns in this regard, with the right education and understanding of these medical conditions, are mandatory in order to increase the transplantation of end-stage diseases.

A formal model based on the automation of the decision process for the management of chronic heart failure, where clinical decisions must be fast and efficient to optimize the treatment of patients before heart transplantation, is a new challenge that the authors want to achieve [70].

## 5. Conclusions

Our retrospective study found a possible impact of chronic HF drug therapy on the early prognosis of heart transplant patients. There were significant negative correlations between the preoperative treatment with Carvedilol and the 2-month mortality rate. Preoperative Ramipril and Spironolactone treatment were also negatively correlated with hospitalization duration, and Ramipril was correlated with ICU stay. Moreover, Carvedilol therapy represented a protective factor against early AKI. Regarding the in-hospital AGR, MRA treatment and Spironolactone were associated with lower event rates, in contrast to ACEI treatment. No statistical correlations were found between the chronic treatment and the complications regardless of cause. Prospective studies, with larger cohorts, are needed in order to draw drastic conclusions.

## Figures and Tables

**Table 1:** Baseline characteristics divided by the presence/absence of complications.

	Total Patients (n = 50)	Complications Regardless of Cause (n = 38)	No Complications (n = 12)	*p* Value
Age, yrs (mean, SD)	41.5 (14.1)	42.2 (12.3)	39.6 (19.4)	0.92 **
Females (n, %)	8 (16.0)	6 (15.8)	2 (16.7)	0.73 ^†^
Weight, kg (mean, SD)	71.6 (20.7)	72.2 (17.3)	69.8 (30.0)	0.73 *
BSA, m^2^ (mean, SD)	1.8 (0.4)	1.9 (0.3)	1.8 (0.5)	0.91 **
BMI, kg/m^2^ (mean, SD)	23.6 (4.9)	23.6 (4.3)	23.5 (6.6)	0.95 *
Rh positive (n, %)	45 (90.0)	34 (89.5)	11 (91.7)	<0.01 ^†^
Blood type O (n, %)	15 (30.0)	11 (28.9)	4 (33.3)	0.48 ^†^
Blood type A (n, %)	25 (50.0)	19 (50.0)	6 (50.0)	1.00 ^†^
Blood type B (n, %)	4 (8.0)	3 (7.9)	1 (8.3)	0.35 ^†^
Blood type AB (n, %)	6 (12.0)	5 (13.2)	1 (8.3)	0.35 ^†^
Ischaemic CM (n, %)	11 (22.0)	8 (21.1)	3 (25.0)	0.48 ^†^
Pre-DM (n, %)	6 (12.0)	4 (10.5)	2 (16.7)	0.35 ^†^
BBs (n, %)	38 (76.0)	30 (78.9)	8 (66.7)	0.78 ^†^
Carvedilol (n, %)	32 (64.0)	24 (63.2)	8 (66.7)	0.83 ^†^
ACEIs (n, %)	19 (38.0)	13 (34.2)	5 (41.7)	0.84 ^†^
Ramipril (n, %)	13 (26.0)	9 (23.7)	2 (16.7)	0.78 ^†^
MRAs (n, %)	46 (92.0)	35 (92.1)	11 (91.7)	0.11 ^†^
Spironolactone (n, %)	41 (82.0)	32 (84.2)	9 (75.0)	1.00 ^†^
Amiodarone (n, %)	26 (52.0)	10 (26.3)	3 (25.0)	0.18 ^†^
Ivabradine (n, %)	19 (38.0)	9 (23.7)	1 (8.3)	0.54 ^†^
Digoxin (n, %)	10 (20.0)	13 (34.2)	6 (50.0)	1.00 ^†^
Hospital stay, days (mean, SD)	57.5 (62.7)	64.0 (70.5)	36.8 (12.4)	0.03 **
Duration of inotrope/vasopressor, days (mean, SD)	8.4 (12.4)	9.4 (13.9)	5.0 (4.3)	0.16 **
ICU stay days (mean, SD)	49.9 (59.2)	56.4 (66.8)	29.5 (4.3)	0.01 **

* Student *t*-test; ** Mann–Whitney U test; ^†^ chi-square test.

**Table 2:** Characteristics of transplanted patients based on the specific chronic HF drug treatment.

	Carvedilol (n = 32)	NO Carvedilol (n = 18)	Ramipril (n = 13)	NO Ramipril (n = 37)	Spironolactone (n = 41)	NO Spironolactone (n = 9)
Daily dose, mg (mean, SD)	19.1 (12.3)		5.2 (2.4)		61.0 (29.1)	
Age, yrs (mean, SD)	40.7 (14.6)	43.0 (13.7)	45.0 (9.0)	40.3 (15.5)	41.6 (13.8)	41.2 16.6
Females (n, %)	6 (18.8)	2 (11.1)	1 (7.7)	7 (18.9)	6 (14.6)	2 (22.2)
Weight, kg (mean, SD)	70.2 (23.2)	74.2 (15.6)	82.8 (15.7)	67.7 (21.0)	71.2 (19.7)	73.4 (26.1)
BSA, m^2^ (mean, SD)	1.8 (0.4)	1.9 (0.2)	2.0 (0.2)	1.8 (0.4)	1.8 (0.3)	1.8 (0.5)
BMI, kg/m^2^ (mean, SD)	23.1 (5.3)	24.5 (4.1)	26.0 (4.2)	22.8 (4.9)	23.4 (4.8)	24.5 (5.4)
Rh positive (n, %)	28 (87.5)	17 (94.4)	12 (92.3)	33 (89.2)	37 (90.2)	8 (88.9)
Blood type O (n, %)	10 (31.3)	5 (27.8)	5 (38.5)	10 (27.0)	13 (31.7)	2 (22.2)
Blood type A (n, %)	17 (53.1)	8 (44.4)	4 (30.8)	21 (56.8)	22 (53.7)	3 (33.3)
Blood type B (n, %)	2 (6.3)	2 (11.1)	3 (23.1)	1 (2.7)	3 (7.3)	1 (11.1)
Blood type AB (n, %)	3 (9.4)	3 (16.7)	1 (7.7)	5 (13.5)	3 (7.3)	3 (33.3)
Ischaemic CM (n, %)	7 (21.9)	4 (22.2)	5 (38.5)	6 (16.2)	9 (22.0)	2 (22.2)
Pre-DM (n, %)	2 (6.3)	4 (22.2)	2 (15.4)	4 (10.8)	4 (9.8)	2 (22.2)
AGR (n, %)	9 (28.1)	2 (11.1)	4 (30.8)	7 (18.9)	6 (14.6)	5 (55.6)
Early AKI (n, %)	14 (43.8)	14 (77.8)	5 (38.5)	23 (62.2)	22 (53.7)	6 (66.7)
Newly diagnosed DM (n, %)	18 (56.3)	12 (66.7)	8 (61.5)	22 (59.5)	26 (63.4)	4 (44.4)
Post-AFib (n, %)	5 (15.6)	5 (27.8)	1 (7.7)	9 (24.3)	8 (19.5)	2 (22.2)
Sepsis (n, %)	5 (15.6)	4 (22.2)	1 (7.7)	8 (21.6)	6 (14.6)	3 (33.3)
Complications regardless of cause (n, %)	24 (75.0)	14 (77.8)	9 (69.2)	29 (78.4)	32 (78.0)	6 (66.7)
Hospital stay, days (mean, SD)	51.5 (34.0)	68.1 (95.0)	40.6 (40.6)	63.4 (68.3)	53.9 (64.8)	73.6 (51.8)
Duration of inotrope/vasopressor, days (mean, SD)	6.1 (5.7)	12.4 (18.9)	5.2 (4.6)	9.5 (14.1)	8.6 (13.5)	7.3 (5.4)
ICU stay days (mean, SD)	41.4 (21.7)	65.0 (94.2)	34.0 (27.1)	55.5 (66.4)	50.3 (64.2)	48.3 (29.8)
2-month mortality (n, %)	1 (3.1)	4 (22.2)	1 (7.7)	4 (10.8)	5 (12.2)	0 (0.0)

**Table 3:** Correlations between drug therapy and the primary outcomes.

Medication	Complications Regardless of Cause R Value (95% CI) *p* Value	Hospital Stay R Value (95% CI) *p* Value	Duration of Inotrope/ Vasopressor R Value (95% CI) *p* Value	ICU Stay R Value (95% CI) *p* Value	2-Month Mortality R Value (95% CI) *p* Value
BBs	0.12 (−0.16–0.38) 0.39	−0.17 (−0.43–0.11) 0.23	−0.04 (−0.31–0.24) 0.78	0.19 (−0.44–0.08) 0.17	0.03 (−0.25–0.30) 0.83
Carvedilol	−0.03 (−0.30–0.25) 0.83	−0.01 (−0.28–0.27) 0.95	−0.10 (−0.37–0.18) 0.47	−0.06 (−0.34–0.21) 0.63	−0.30 (−0.53–−0.02) 0.03
ACEIs	−0.13 (−0.40–0.14) 0.33	−0.20 (−0.45–0.08) 0.15	0.03 (−0.25–0.30) 0.84	−0.18 (−0.44–0.09) 0.19	−0.12 (−0.39–0.16) 0.39
Ramipril	−0.09 (−0.36–0.19) 0.51	−0.38 (−0.60–−0.12) <0.01	−0.15 (−0.41–0.13) 0.30	−0.37 (−0.59–−0.11) 0.01	−0.04 (−0.32–0.23) 0.75
MRAs	0.01 (−0.27–0.28) 0.96	−0.21 (−0.46–0.06) 0.13	−0.17 (−0.43–0.11) 0.24	−0.22 (−0.47–0.05) 0.11	0.09 (−0.18–0.36) 0.49
Spironolactone	0.10 (−0.18–0.37) 0.47	−0.28 (−0.52–−0.01) 0.04	−0.16 (−0.42–0.12) 0.25	−0.14 (−0.40–0.14) 0.32	0.15 (−0.12–0.41) 0.27

**Table 4:** Associations between drug therapy and secondary outcomes.

Medication	AGR OR (95% CI) *p* Value	Early AKI OR (95% CI) *p* Value	Newly Diagnosed DM OR (95% CI) *p* Value	AFib OR (95% CI) *p* Value	AGR OR (95% CI) *p* Value
BBs	1.67 (0.29–9.50) 0.56	0.54 (0.12–2.29) 0.40	1.42 (0.34–5.91) 0.62	0.68 (0.13–3.53) 0.65	1.67 (0.29–9.50) 0.56
Carvedilol	3.92 (0.64–24.02) 0.13	0.22 (0.05–0.91) 0.03	1.07 (0.27–4.19) 0.92	0.54 (0.11–2.63) 0.45	3.92 (0.64–24.02) 0.13
ACEIs	5.30 (1.03–27.17) 0.04	0.34 (0.09–1.31) 0.11	0.39 (0.10–1.46) 0.16	0.39 (0.05–2.63) 0.33	5.30 (1.03–27.17) 0.04
Ramipril	2.18 (0.47–10.13) 0.31	0.40 (0.10–1.66) 0.21	1.69 (0.41–7.01) 0.46	0.27 (0.03–2.66) 0.26	2.18 (0.47–10.13) 0.31
MRAs	0.05 (0.01–0.67) 0.02	0.30 (0.01–5.00) 0.40	0.40 (0.03–5.53) 0.49	2.01 (0.10–37.11) 0.63	0.05 (0.01–0.67) 0.02
Spironolactone	0.12 (0.02–0.66) 0.01	0.35 (0.05–2.22) 0.26	2.78 (0.50–15.44) 0.24	1.24 (0.14–11.09) 0.84	0.12 (0.02–0.66) 0.01

## Data Availability

The data that support the findings of this study are available from the corresponding author upon reasonable request.
